# Lactide-Valerolactone Copolymers for Packaging Applications

**DOI:** 10.3390/polym14010052

**Published:** 2021-12-23

**Authors:** Ainara Sangroniz, Leire Sangroniz, Shaghayegh Hamzehlou, Nora Aranburu, Haritz Sardon, Jose Ramon Sarasua, Marian Iriarte, Jose Ramon Leiza, Agustin Etxeberria

**Affiliations:** 1 POLYMAT, Department of Advanced Polymers and Materials: Physics, Chemistry and Technology, Faculty of Chemistry, University of the Basque Country UPV/EHU, Manuel de Lardizabal 3 Pasealekua, 20018 Donostia, Spain; leire.sangroniz@ehu.es (L.S.); nora.aramburu@ehu.eus (N.A.); haritz.sardon@ehu.eus (H.S.); marian.iriarte@ehu.eus (M.I.); 2 POLYMAT, Department of Applied Chemistry, Faculty of Chemistry, University of the Basque Country UPV/EHU, Joxe Mari Korta Zentroa, Tolosa Hiribidea 72, 20018 Donostia, Spain; shaghayegh.hamzehlou@ehu.eus (S.H.); jrleiza@ehu.eus (J.R.L.); 3 POLYMAT, Department of Mining-Metallurgy Engineering and Materials Science, University of the Basque Country UPV/EHU, Torres Quevedo Ingeniariaren Plaza 1, 48013 Bilbao, Spain; iipsaoij@ehu.es

**Keywords:** polylactide, biodegradable, permeability, packaging

## Abstract

Lactide-valerolactone copolymers have potential application in the packaging sector. Different copolymers were synthesized, and the kinetics of the copolymerization reactions and the microstructure of the copolymers were analysed. Lactide showed higher reactivity than valerolactone which leads to composition drift through the reaction. Thermal, mechanical and barrier properties of the selected copolymers were studied. Overall, the incorporation of valerolactone results in copolymers with higher ductility than poly(lactide) with intermediate water and oxygen permeability which makes these materials appropriate candidates for use in the packaging sector.

## 1. Introduction

Nowadays the materials used on the market, such as polyolefins ((poly(ethylene) and poly(propylene)) and poly(ethylene terephthalate), are contributing to the problem of plastic waste since they are obtained from petroleum, and they cannot be degraded [1,2,3,4,5].

Bearing in mind these issues, in recent years, biodegradable polymers have attracted great attention since they can be degraded to biomass, carbon dioxide, water and similar substances, and no waste is generated [1,2,3,4]. Therefore, they are the ideal candidates to replace conventional polymers for example in packaging applications, bags or mulch films, among others.

Among biodegradable polymers, one of the most important is poly(lactide) (PLA). PLA has been widely studied in the literature since it is biocompatible and can be employed in scaffolds, stents, catheters and controlled-release drug carriers, among others [6,7,8,9]. However, PLA shows a brittle behaviour which prevents it from being used in a wide range of applications. In order to improve its ductility, different approaches have been analysed, one of them being to copolymerize it with another comonomer which leads to a material with a high elongation at break [7,8,10,11].

Lactide based copolymers based on caprolactone, valerolactone, decalactone, ethylene brassylate and other lactones have been synthetized, and their degradation has been widely studied [12,13,14,15,16,17]. These materials could have also a potential application in packaging, and for that, the barrier character of the materials must be studied. However, the works addressing this issue are scarce, and only one reference [11] has considered this point.

In a previous work, we studied the suitability of lactide-caprolactone copolymers for packaging applications [11]. In this work, we have selected valerolactone that can lead to polymers with lower glass transition temperature than PLA but higher than lactide-caprolactone copolymer, better stress related properties (such as higher Young modulus and yield strength) and a lower degradation rate compared to lactide-caprolactone copolymers [10] which could be interesting for packaging applications. Furthermore, in the literature, the enzymatic and hydrolytic degradation of lactide-valerolactone copolymers has been studied. Both the enzymatic degradation employing a lipase from *Rhizopus arrhizus* and the hydrolytic degradation show that copolymers can be hydrolysed [12]. Thus, this copolymer system could be a good candidate for packaging applications.

In this work, first, the kinetics of the reaction and the microstructure of the copolymers were studied. Second, for selected copolymers, the physico-chemical properties were studied, focusing on permeability to gases and vapours and the mechanical properties in order to analyse the suitability of these materials in packaging applications.

## 2. Materials and Methods

### 2.1. Materials and the Synthesis Procedure of Copolymers

#### 2.1.1. Materials

L-lactide (Futerro, Hainaut, Belgium) was recrystallized from dried toluene. δ-valerolactone (Sigma Aldrich, Burlington, MA, USA) was distilled prior to use. As a catalyst, triphenyl bismuth was employed, which was provided by Gelest Inc. (Morrisville, PA, USA) and was used as received. The purification of monomers and the synthesis of the copolymer were carried out in a high vacuum line or in a glovebox filled with inert gas.

#### 2.1.2. Synthesis Procedure of the Copolymers

The copolymerization reactions for the kinetic analysis were performed at 140 °C and 100:1 monomer:catalyst ratio. The reactions were performed in 0.5 g scale in small glass vials. After a certain time, aliquots were taken and analysed by ^1^H NMR.

The copolymerization reactions in large scale (10 g) were carried out at 140 °C for 24 h and at 100:1 monomer:catalyst ratio. The obtained polymers were dissolved in chloroform and precipitated in cold methanol to purify them. Afterwards, the materials were dried at 70 °C for 48 h.

#### 2.1.3. Film Preparation

The films were prepared using a hot-press at 190 °C. Films with thickness in the range 100–150 μm were obtained. The obtained membranes were dried for 2 days under vacuum at 70 °C. Subsequently, the membranes were dried for at least 4 days at room temperature under vacuum.

### 2.2. Techniques and Instruments

#### 2.2.1. NMR Measurements

The spectra were recorded on a Bruker Avance DPX 300 spectrometer (Billerica, MA, USA) at 300.16 MHz for proton spectra and at 75.5 MHz for carbon spectra. Tubes with an internal diameter of 5 mm were employed, and the measurements were performed at 30 °C. For proton NMR, 10 mg of sample were dissolved in 0.4 mL deuterated chloroform, and 32 scans were performed. In the case of carbon spectra, 40 mg of sample were dissolved in 0.4 mL of deuterated chloroform, and more than 5000 scans were performed.

#### 2.2.2. Molar Mass Characterization

Size exclusion chromatography was employed to determine the molar mass of samples using a Waters 1515 GPC device (Milford, MA, USA) with two Styragel columns (10^2^–10^4^ Å). The flow of chloroform was 1 mL min^−1^, and the molar masses were referred to poly(styrene) standards.

#### 2.2.3. Thermal Analysis

Thermal properties were characterized employing a differential scanning calorymeter Q2000 V24 (TA Instrument, New Castle, DE, USA). Moreover, 3–5 mg of the samples were encapsulated in aluminium pans, and two scans were performed from −80 °C to 200 °C at 10 °C min^−1^ heating and cooling rate.

In order to determine the crystallinity degree of the copolymers, the following equation was employed:

(1) $$X_{c}=\frac{∆H_{m}}{∆H_{m}^{0}}\times 100$$

where $∆H_{m}\;$ is the experimental value of the melting enthalpy obtained from the DSC experiments, and $∆H_{m}^{0}$ is the theoretical value of the melting enthalpy of 100% crystalline PLLA with a value of 106 J g^−1^ [18].

#### 2.2.4. Thermogravimetric Analysis

TGA Q 500 equipment (TA Instrument, New Castle, DE, USA) was employed to perform the thermal gravimetric analysis. In addition, 3 mg samples were heated from 25 °C to 800 °C at a heating rate of 10 °C min^−1^ under nitrogen flow, 100 mL min^−1^.

#### 2.2.5. Characterization of Mechanical Properties

The mechanical properties were measured in an Instron 5565 testing machine (Norwood, MA, USA) at a crosshead displacement rate of 5–100 mm/min, depending on the sample, and 22 °C. The films had a thickness between 100 and 150 μm, and the specimens were cut according to ASTM D638 type V. At least 5 specimens were tested for each reported value.

#### 2.2.6. Characterization of Transport Properties

The water vapour transmission rate was measured following the ASTM E96-95 gravimetric method. The cell employed to measure the permeability is a small container made of polytetrafluoroethylene, which is partially filled with water and a polymeric membrane at the top. The measurements were carried out at 25 °C in a balance (Sartorius BP 210 D, Goettingen, Germany) with 10^−5^ g readability, and the mass loss was recorded in a computer. The values shown herein are the average of at least 5 measurements.

The oxygen permeability coefficient was measured with a Mocon OX-TRAN 2/21 MH model equipment (Mocon Inc., Brooklyn Park, MN, USA) at 1 atm, 30 °C and 0% relative humidity. Further details about the equipment and measurement methods can be found elsewhere [18,19,20,21,22].

## 3. Results

### 3.1. Characterization of the Chain Microstructure of LA-VL Copolymers

**Figure 1:**
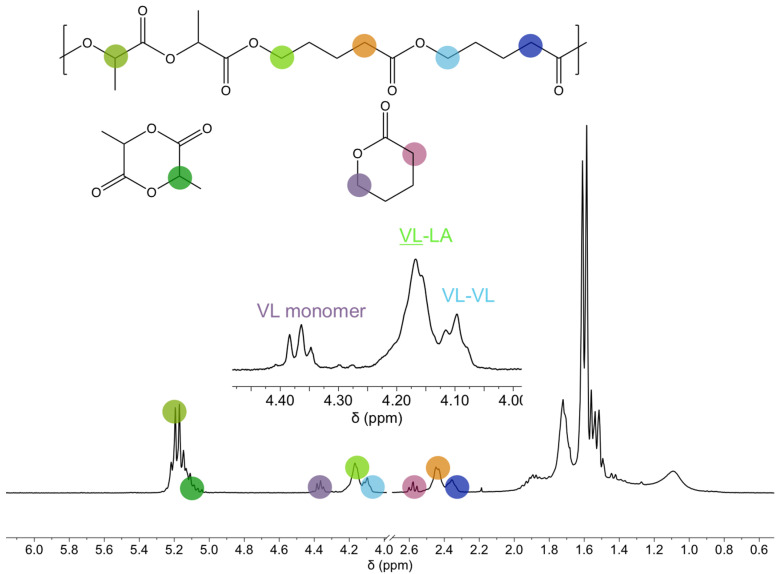
1H NMR spectra of 50 LA 50 VL copolymer after 180 min reaction.

In this section, the chain microstructure of the copolymers and the evolution of this microstructure during the polymerization reaction are addressed. For that, the 1H NMR of one of the copolymers is discussed first, as an example, to show the signals employed to calculate the monomer conversion and to characterize the microstructure of the copolymers. In Figure 1, the 1H NMR corresponding to 50 LA 50 VL copolymer after 180 min reaction time is shown. The following signals have been considered: the signals at 5.17 ppm and 5.04 ppm correspond to the methine of the lactide copolymer and the monomer, respectively. The signals at 4.36 ppm, 4.17 ppm and 4.10 ppm correspond to the methylene next to OCO of valerolactone monomer, VL-LA dyad and VL-VL dyad, respectively. The signal at 2.58 ppm corresponds to the methylene next to COO, the signal at 2.45 ppm to the VL-LA dyad and the one at 2.36 ppm to the VL-VL dyad. In the range of 1.0–2.0 ppm, the signals corresponding to the rest of the methylenes of valerolactone and the methyl of lactide appear.

**Figure 2:**
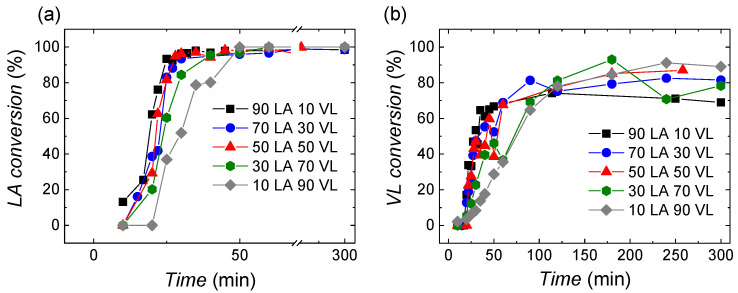
Monomer conversion evolution of (**a**) lactide and (**b**) valerolactone.

The conversion of lactide and valerolactone monomers at different monomer composition feeds has been followed by 1H NMR spectroscopy, and the data are shown in Figure 2a,b. The obtained results show that the polymerization of lactide is very fast (see Figure 2a), especially in the case of the reactions where the monomer feeds are rich in lactide. The copolymer containing the smallest amount of lactide, 10 LA 90 VL, shows a slower polymerization rate. In all the reactions, full conversion of lactide is achieved in less than 50 min.

Regarding valerolactone (Figure 2b), after 300 min of reaction, the monomer does not reach full conversion due to its lower reactivity ratio and because of the low (homo) propagation rate constant. The conversion values obtained are higher than 70%. As in the case of lactide, the polymerization rate depends on the monomer feed ratio. Thus, for monomer feeds rich on lactide, the polymerization of valerolactone is faster than for those that are rich in valerolactone.

In order to gain further insight on the chain microstructure evolution during the reaction, the number-average sequence length of lactide (*l_LA_*) and valerolactone (*l_VL_*) as well as the randomness character (*η*) were calculated. For that purpose, Equations (2)–(4) have been employed [23].

(2) $$l_{LA}=\frac{2\left(LA\right)}{\left(LA-VL\right)}$$

(3) $$l_{VL}=\frac{2\left(VL\right)}{\left(LA-VL\right)}$$

(4) $$\eta =\frac{\left(LA-VL\right)}{2\left(LA\right)\left(VL\right)}$$

where (*LA*) is the lactide molar fraction; (*VL*) is the valerolactone molar fraction and (*LA* − *VL*) is the *LA* − *VL* average dyad relative molar fraction. More details about calculations can be found elsewhere [11].

**Figure 3:**
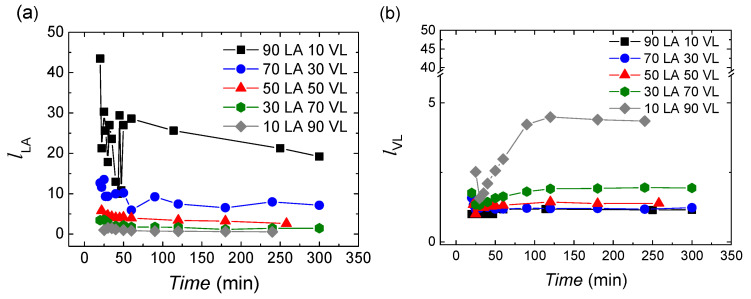
The number-average sequence lengths of (**a**) lactide and (**b**) valerolactone.

The lactide sequence length varies with the monomer feeding and reaction time. In Figure 3a, it can be seen that the largest lactide sequence length is obtained for the reactions with monomer feedings rich in lactide, and as the content of valerolactone increases, the sequence length is reduced. The values are in the range of 40.8–0.6. During the reaction, the lactide sequence length is decreased; this arises probably from the higher affinity of lactide with lactide than valerolactone. When lactide monomer is consumed valerolactone is incorporated to the chains.

In the case of valerolactone, the number-average sequence lengths are shorter than the length of lactide with values in the range of 1–5. As the content on valerolactone in the feeding raises, the sequence length of valerolactone increases, as expected.

It has to be taken into account that the calculated length of the sequence is a cumulative average value. In the first 50 min, practically all the lactide is incorporated while only 73.5–31.7% VL has reacted. After 50 min, as practically 100% of lactide is consumed, valerolactone would be consumed alone. Thus, two options are possible for its consumption: (1) valerolactone units are incorporated in copolymer chains generating gradient type copolymers, or (2) valerolactone units are incorporated in new chains obtaining VL homopolymer. Furthermore, it has to be considered that after 50 min, all the lactide is consumed, so the observed reduction of the *l_LA_* sequence length could come only from transesterification reactions (option 1). Anyway, the possible formation of the VL homopolymer will be discussed later.

**Figure 4:**
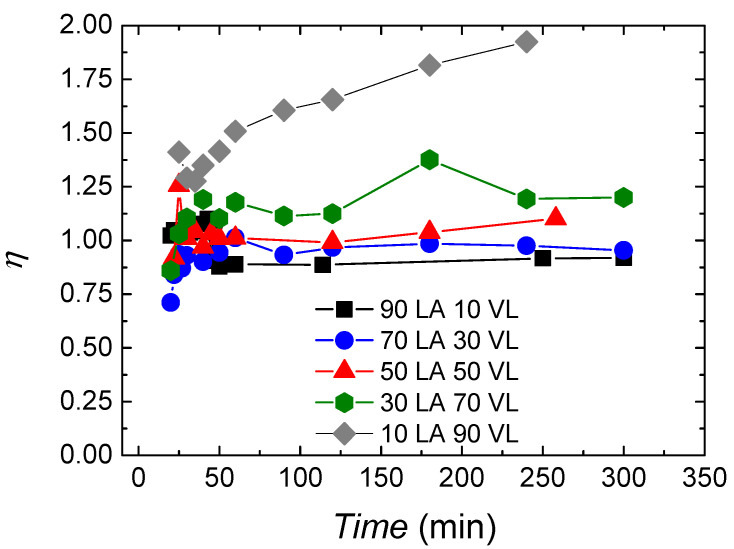
Randomness character evolution during reaction for lactide-valerolactone copolymers.

In Figure 4, the randomness character of the copolymers is shown, which allows to analyse the microstructure of the copolymers in further detail. At the beginning of the reaction, the randomness character is about 1 for all the copolymers, showing a random character. During the reaction, the randomness character oscillates between 0.8–1.25. Moreover, 10 LA 90 VL shows a different behaviour with values around 1.25 at the beginning of the reaction and by achieving a value of 2 at the end of the reaction.

The randomness character does not show the real nature of the copolymers. Taking into account the monomer conversion data and the sequence length of the monomers over time, it can be forecasted that big lactide blocks are formed at the beginning, and afterwards, valerolactone is introduced in the existing polymer chains or in new polymer chains; thus, copolymers with gradient like structure or valerolactone homopolymers are obtained. In any case, further studies are being carried out in order to clarify this.

As mentioned above, the 10 LA 90 VL copolymer shows a different behaviour with a value of 1.4 at the beginning that increases gradually, reaching values near 2 by the end of the reaction, which is indicative of alternant character. In this case, since the lactide has already polymerized when valerolactone starts to react, the change of the randomness character of the copolymer with the reaction time could arise from transesterification reactions that have been corroborated by ^13^C NMR; see Figure S1 in Supplementary Information. In fact, a peak appears at 170.6 ppm for copolymers with 19% valerolactone content or higher corresponding to VL-LA-VL sequence. A similar peak appears in lactide-caprolactone copolymers at 171 ppm corresponding to 171 ppm. For the other copolymers, also transesterification could be expected, but taking into account that they are rich on lactide and that transesterification would occur between lactide–lactide units, it cannot be accurately quantified by NMR. In the case of 30 LA 70 VL, also a slight tendency to form alternant like copolymers is observed.

To further study the copolymerization reactions, the reactivity ratios of lactide and valerolactone were calculated employing a nonlinear parameter estimation algorithm [24]; see Table 1. The algorithm determines the reactivity ratios by minimizing the prediction of a model based on the Mayo–Lewis equation and the experimental data gathered by NMR for the cumulative copolymer composition. For more details about the estimation algorithm, see Note S1 in Supplementary Information. It can be seen that lactide presents a higher reactivity ratio than valerolactone, being 47 times higher, which could be also deduced from the faster monomer conversion of lactide than valerolactone observed in Figure 2.

**Figure 5:**
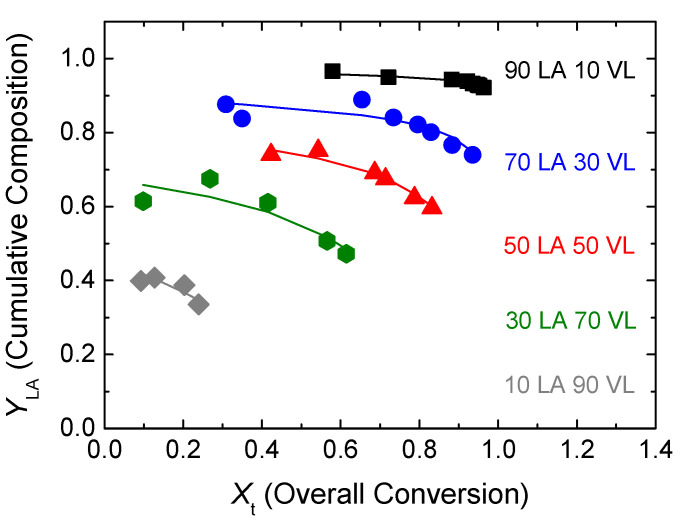
Conversion evolution of the cumulative composition of LA. Experimental results (dots) and model predictions (lines) for the estimated reactivity ratios.

In Figure 5, the comparison between the cumulative copolymer composition of lactide determined experimentally by NMR analysis (dots) and the calculated cumulative composition (lines) using the estimated reactivity ratios is shown. The fitting of the cumulative composition for the experiments carried out at different monomer feeds is reasonable in all the reactions.

### 3.2. Physico-Chemical Characterization of Selected Copolymers for Packaging Applications

In order to analyse the suitability of lactide-valerolactone copolymers in packaging applications, copolymers rich in lactide were selected, since lowering the glass transition temperature could lead to a higher ductility while maintaining the stress related properties (Young modulus and yield stress). Thus, copolymers were prepared in a large scale, and their physico-chemical properties have been studied.

The randomness character and the number-average sequence length of lactide and valerolactone were calculated from ^1^H NMR spectra; see Table S1 in Supporting Information. All the copolymers show a randomness character near 1, and therefore, random copolymers are obtained which was the goal of this work.

#### 3.2.1. Molar Mass Characterization

The molar mass was determined by Size Exclusion Chromatography, and the values were calculated by GPC relative to poly(styrene) standards; the data are shown in Table 2. PLLA shows a molar mass of 256.3 kg/mol and a dispersity index of 2.4. In the case of copolymers, the incorporation of VL decreases the molecular weight, likely due to the lower overall polymerization rate caused by the lower rate of propagation of valerolactone. The broad dispersity index obtained in all the cases can be attributed to the transesterification reactions, which have been detected in our copolymers from NMR spectra.

In any case, the GPC data could become clear if VL incorporates rich growing chains to the LA or if it forms new chains generating VL homopolymer chains. In this case, taking into account the lower amount of VL, a broad signal or a signal with a shoulder at lower molar weight would be observed or even a bimodal molecular weight distribution, but neither of both cases are observed. It could also be possible that the VL homopolymer amount is so small that is not possible to discern it in the GPC chromatogram. Therefore, we can deduce that the initial structure of the copolymers is gradient type one, but the transesterification reactions gradually modify such structure towards a more random nature.

#### 3.2.2. Thermal Properties Characterization

The thermal properties play an important role on the transport properties; therefore, they were analysed by DSC; see Table 3. The glass transition temperature was characterized in the second DSC heating scan. PLLA shows a glass transition temperature of 55 °C. In the case of copolymers, as the content on valerolactone increases, the glass transition temperature is decreased gradually. It is worth noting that the copolymers with valerolactone content higher than 33% show an elastomeric behaviour. This is important since the transport properties and, particularly, sorption process is different in glassy and elastomeric polymers.

The glass transition temperature was theoretically calculated employing the additive rule:

(5) $$T_{g}=T_{g1}w_{1}+T_{g2}w_{2}$$

where *T*_*g*_ is the theoretical glass transition temperature, *T*_*g*1_ and *T*_*g*2_ are the glass transition temperatures of PLLA and PVL, and *w*_1_ and *w*_2_ are the weight fractions of each polymer. In the case of PVL, *T*_*g*_ = −67 °C [25].

Another model employed to calculate theoretically the glass transition temperature is the Fox model [26,27]:

(6) $$\frac{1}{T_{g}}=\frac{w_{1}}{T_{g1}}+\frac{w_{2}}{T_{g2}}$$

**Figure 6:**
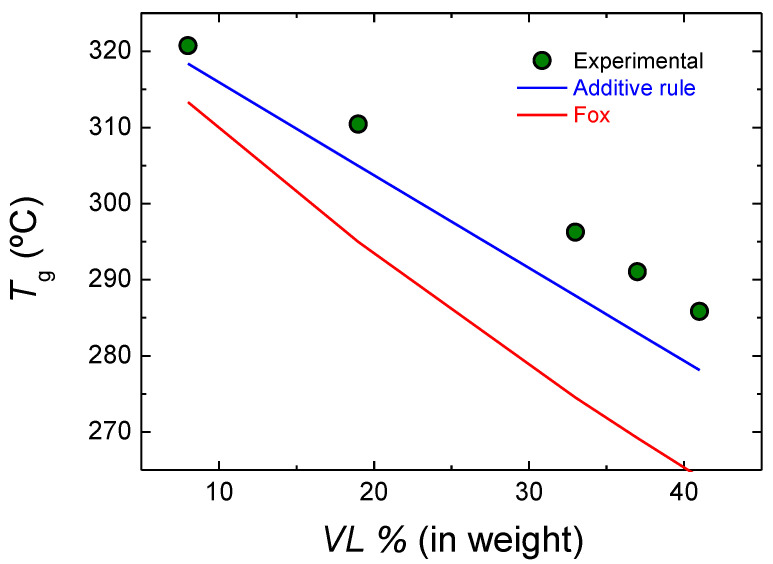
Experimental and theoretical glass transition temperatures of lactide-valerolactone copolymers.

The experimental glass transition temperatures and those calculated theoretically are depicted in Figure 6. The experimental results are slightly higher than the values obtained with the additive rule. On the other hand, the Fox model underestimates the glass transition temperature values obtained. This could arise from the fact that the Fox model describes properly the glass transition temperature of ideal Bernouillian random copolymers, and our copolymers deviate from this model due to both gradient character and the transesterification reactions.

The melting temperature and the crystallinity level of the samples were obtained from the first DSC heating scan. PLLA shows a melting temperature of 177.0 °C, and for the copolymers, as the content on valerolactone rises, the melting temperature is decreased. In the case of the copolymer containing 41% VL, the sample is amorphous. The obtained results are expected since the incorporation of a second monomer reduces the crystallisable chain length segment of lactide leading to the reduction of the melting temperature.

Employing the following equation, the melting temperatures of the copolymers have been theoretically calculated:

(7) $$∆H_{m}^{0}\left(\frac{1}{T_{m}}-\frac{1}{T_{m}^{0}}\right)=-R\cdot \ln X$$

where $∆H_{m}^{0}$ is the theoretical value of the melting enthalpy of 100% homocrystalline PLLA that has a value of 106 J/g, $T_{m}$ is the melting temperature of the copolymer, $T_{m}^{0}$ is the melting temperature of PLLA ($T_{m}^{0}\;$= 450 K), and X is the molar fraction of lactide [28].

**Figure 7:**
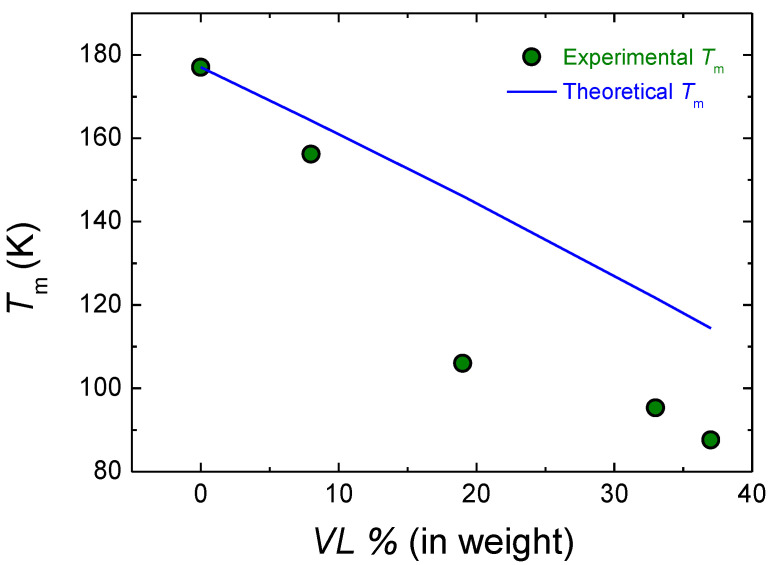
Experimental and theoretical melting temperature of LA-VL copolymers.

In Figure 7, the experimental melting temperature and the theoretical melting temperature obtained with Equation (5) are shown. The theoretical model overestimates the melting temperature of the copolymers except in the case of the 92 LA 8 VL copolymer, where the theoretical melting temperature is closer to the experimental value. The difference between the theoretical model and the experimental results could arise from the gradient-type nature of the copolymer or the transesterification reactions.

Taking into account that the crystallites are considered impermeable to gases and vapours, the characterization of the crystallinity level is of paramount importance. PLLA shows a crystallinity level of 34%, and in the case of copolymers, as the content on valerolactone rises, the crystallinity level decreases gradually. For the copolymers containing more than 33% of valerolactone, the crystallinity level is very small, almost negligible.

Returning to the question of VL incorporation mode, if VL homopolymer is obtained, it will be reasonable to observe the corresponding *T_g_* of pure VL and even the formation of VL crystalline phase, but none of these facts are observed. It could also be possible that the small amount of VL homopolymer cannot be noticed in the DSC scan. Moreover, as mentioned above, the *T_g_* of the copolymer decreases gradually with the content of VL. Thus, it can be concluded that the initial structure of the LA-VL copolymer would be gradient type one, but transesterification reactions modify largely such structure leading to a copolymer with a random nature.

The thermal stability of the copolymers was studied by a thermogravimeter analyser in order to investigate if these materials could be processed by film blown extrusion.

The obtained data are shown in Table S2 in Supplementary Information. PLLA shows a T_5%_ of 211 °C (temperature at which 5% of weight is loss), and in the case of the copolymers, slightly higher values are obtained in the range of 214–224 °C, so they show a thermal stability higher than PLLA. Therefore, selecting the appropriate processing temperature, these copolymers could be processed by film blown extrusion.

#### 3.2.3. Mechanical Properties

**Figure 8:**
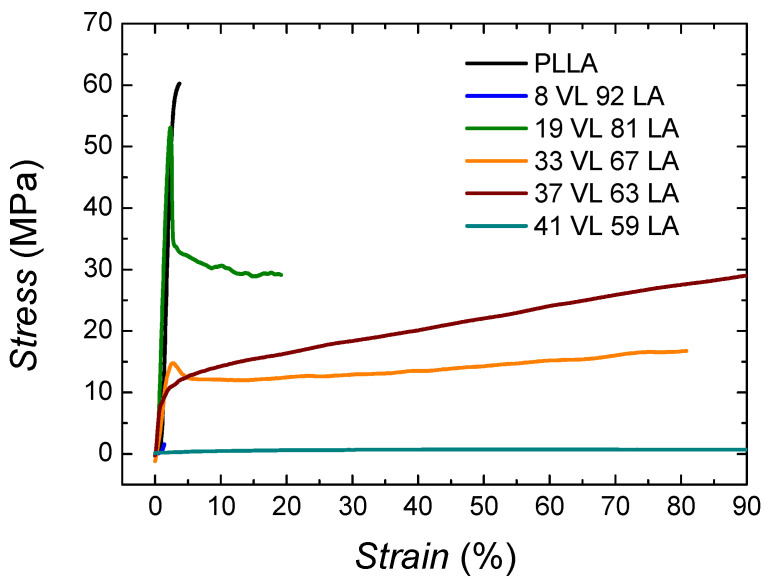
Stress–strain curves of lactide-valerolactone copolymers. The graph was cut along the x axis to better observe the initial part of the curves.

In order to assess the suitability of these materials for packaging applications, it is necessary to characterize the mechanical properties, and for that purpose, tensile tests were performed. In Figure 8, the stress–strain curves of PLLA and lactide-valerolactone copolymers are depicted.

The behaviour of PLLA corresponds to a stiff and brittle polymer, showing high modulus (3390 MPa) and low ductility (3.8%). In the case of the copolymers, as the content on valerolactone increases, the materials show an elastomeric behaviour resembling a soft material.

**Figure 9:**
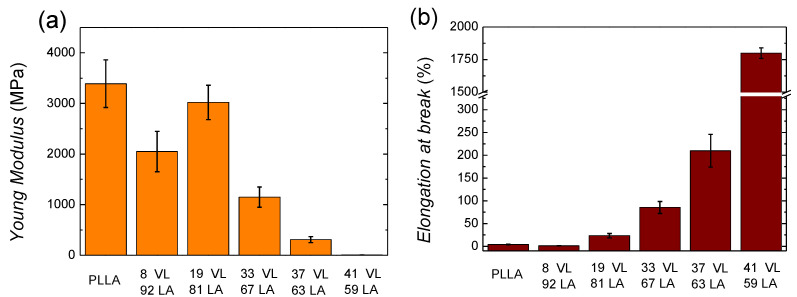
Young modulus (**a**) and elongation at break (**b**) of lactide-valerolactone copolymers.

Figure 9a shows the Young modulus and Figure 9b the elongation at break of lactide-valerolactone copolymers. The data can be found in Table S3 in Supplementary Information. As it can be seen in Figure 9a, the incorporation of valerolactone decreases the Young modulus considerably, probably due to the lower glass transition temperature and crystallinity level or the lack of it.

Regarding the tensile strength, the incorporation of valerolactone decreases the ultimate strength gradually (see Table S3 in Supporting Information), as it could be expected since the glass transition temperature and the crystallinity level are decreased. In the case of the copolymer containing 8% VL, the values of the three magnitudes are considerably lower than expected. As mentioned above, a similar behaviour has been obtained for the molecular weight. This larger decrease of the molecular weight would lead to an anomalous stiffness and a brittle behaviour.

The elongation at break (see Figure 9b and Table S3 in Supplementary Information) increases gradually with the valerolactone content. In the case of the copolymer containing 33% VL, a significant increase is found with a value of 85%. The 41 VL 59 LA copolymer shows a high elongation at break, 1800%, showing elastomeric behaviour.

#### 3.2.4. Barrier Properties

The barrier properties of the copolymers have been studied in order to analyse the suitability of these materials in the packaging applications.

Water vapour transmission rate was characterized since water can cause chemical and physical changes on the packaged product, and it can promote the growth of microorganisms that deteriorate food [4,29].

**Figure 10:**
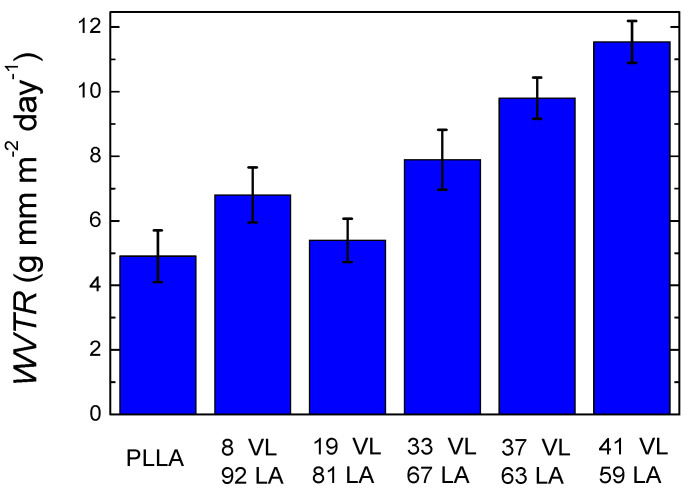
Water vapour transmission rate of lactide-valerolactone copolymers.

Figure 10 shows the water vapour transmission rate of lactide-valerolactone copolymers (the data are shown in Table S4 in Supplementary Information). PLLA shows a value of 4.9 g mm m*^−^*^2^ day^−1^, which is similar to the data reported in the literature [20]. In the case of copolymers, the incorporation of valerolactone gradually increases the water vapour transmission rate. The copolymers with the lowest and highest valerolactone content show the following water vapour transmission rates: 6.8 g mm m^−2^ day^−1^ for the copolymer with 8% VL and 11.5 g mm m*^−^*^2^ day*^−^*^1^ for the copolymer containing 41% VL. The increase of WVTR with the increment of valerolactone in the copolymer could arise from the lower glass transition temperature together with the lower crystallinity level or the lack of it.

A clear trend is observed in the water vapour transmission rate with the incorporation of valerolactone except for 8% VL. The results obtained for 8 VL 92 LA cannot be explained by the glass transition temperature or by the crystallinity level. Therefore, it is possible that the free volume fraction and the interactions between water and the copolymers lead to this result [30].

These results show a different trend compared to lactide-caprolactone copolymers, where the transmission rate of the copolymers was lower than neat polylactide. This will be discussed below.

It is also interesting to measure oxygen permeability since oxygen can provoke oxidative reactions causing rancid flavours and colour and nutritive changes. Oxygen also enables the growth of aerobic microorganisms [29].

**Figure 11:**
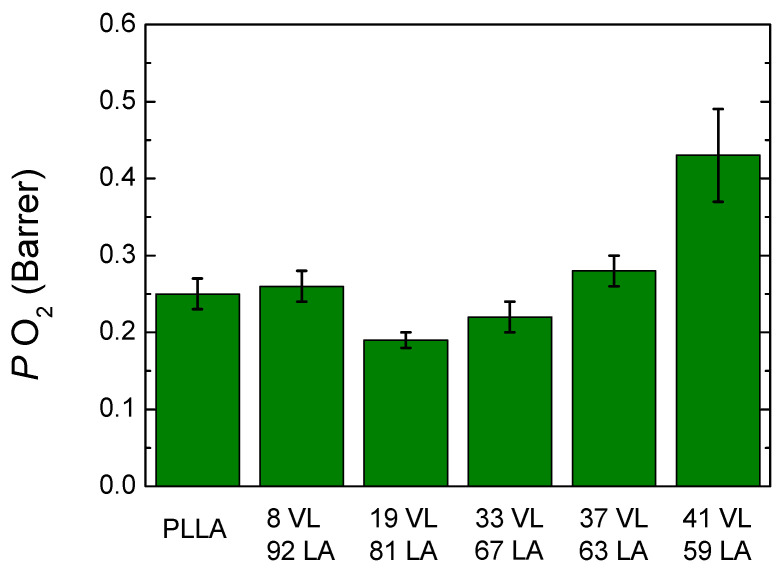
Oxygen permeability of lactide-valerolactone copolymers.

Figure 11 shows the oxygen permeability of lactide-valerolactone copolymers (the data are shown in Table S4 in Supplementary Information). PLLA shows a value of 0.25 Barrer, which is similar to those reported in literature; 8 VL 92 LA copolymer shows a slightly higher permeability, 0.26 Barrer, which is the expected result taking into account its lower glass transition temperature. The copolymers containing 19–33% valerolactone show a lower oxygen permeability than PLLA which is an unexpected result taking into account that the glass transition temperature is lower than PLLA, and the crystallinity level is also reduced. Furthermore, the results obtained are different from those obtained for the lactide-caprolactone system, where an increase of the permeability is found with the incorporation of caprolactone. Furthermore, lactide-valerolactone copolymers show lower permeability values. These results could arise from the delicate balance between interactions, free volume and size of penetrants, among others.

## 4. Conclusions

Lactide-valerolactone copolymers were synthetized for packaging applications. First, the polymerization kinetics and microstructure of the copolymers were studied, and then, for selected copolymers, the thermal, mechanical and barrier properties were studied.

The polymerization kinetics shows that lactide polymerizes faster than lactide, obtaining gradient like copolymers which afterwards are largely modified by transesterification reactions. Therefore, copolymers with a randomness character are obtained.

Regarding the physico-chemical characterization, the incorporation of valerolactone decreases the glass transition temperature which indicates that materials with a higher ductility are obtained, as corroborated by stress–strain measurements. The water vapour transmission rate increases with the incorporation of valerolactone, whereas the oxygen permeability is decreased slightly for copolymers containing small valerolactone amounts. Thus, depending on the final application, tailored copolymers can be obtained that have the appropriate mechanical and barrier properties.

## Figures and Tables

**Table 1:** Reactivity ratios of lactide and valerolactone.

*r*_LA_	*r*_VL_
3.31 ± 0.36	0.07 ± 0.02

**Table 2:** Weight-average molar mass and dispersity index of the homopolymer and copolymers.

VL%	*M*_w_ (kg/mol)	*D*
0	256.3	2.4
8	86.0	2.8
19	139.3	2.5
33	84.3	2.7
37	59.9	2.6
41	62.8	2.3

**Table 3:** Thermal properties of PLLA and lactide-valerolactone copolymers.

Sample	*T*_*g*_ (°C) 2nd scan	*T*_m_ (°C)	Δ*H*_m_ (J/g)	*X*c (%)	*X*c LA (%)
PLLA	55	177.0	36	34	34
8 VL	48	156.2	36	34	37
19 VL	37	78.1, 106.0	19	18	22
33 VL	23	95.3	1	1	1
37 VL	18	87.6	2	2	3
41 VL	13	-	-	-	-

## Data Availability

The data presented in this study are available in the article and in the Supplementary Materials.

## Supplementary Materials

The following are available online at https://www.mdpi.com/article/10.3390/polym14010052/s1. Figure S1: ^13^C NMR of lactide-valerolactone copolymers. Note S1: Estimation of the monomer reactivity ratios. Table S1: Microstructure parameter of lactide-valerolactone copolymers. Table S2: Thermal degradation of lactide-valerolactone copolymers. Table S3: Mechanical properties of lactide-valerolactone copolymers. Table S4: Water vapour transmission rate and oxygen permeability.

## References

[1] Amass W., Amass A., Tighe B. (1998). A review of biodegradable polymers: Uses, current developments in the synthesis and characterization of biodegradable polyesters, blends of biodegradable polymers and recent advances in biodegradation studies. Polym. Int..

[2] Vroman I., Tighzert L. (2009). Biodegradable polymers. Materials.

[3] Gross A., Kalra B. (2002). Biodegradable polymers for the environment. Green Chem..

[4] Siracusa V., Rocculi P., Romani S., Rosa M.D. (2008). Biodegradable polymers for food packaging: A review. Trends Food Sci. Technol..

[5] Sangroniz A., Zhu J.B., Tang X., Etxeberria A., Chen E.Y.X., Sardon H. (2019). Packaging materials with desired mechanical and barrier properties and full chemical recyclability. Nat. Commun..

[6] Auras R., Harte B., Selke S. (2004). An overview of polylactides as packaging materials. Macromol. Biosci..

[7] Gupta A.P., Kumar V. (2007). New emerging trends in synthetic biodegradable polymers—Polylactide: A critique. Eur. Polym. J..

[8] Nampoothiri K.M., Nair N.R., John R.P. (2010). An overview of the recent developments in polylactide (PLA) research. Bioresour. Technol..

[9] Sonchaeng U., Iñiguez-Franco F., Auras R., Selke S., Rubino M., Limb L.T. (2018). Poly(lactic acid) mass transfer properties. Prog. Polym. Sci..

[10] Fernández J., Etxeberria A., Sarasua J.R. (2015). In vitro degradation of poly(lactide/δ-valerolactone) copolymers. Polym. Degrad. Stab..

[11] Sangroniz A., Sangroniz L., Hamzehlou S., Del Río J., Santamaría A., Sarasua J.R., Iriarte M., Leiza J.R., Etxeberria A. (2020). Lactide-caprolactone copolymers with tuneable barrier properties for packaging applications. Polymer.

[12] Nakayama A., Kawasaki N., Maeda Y., Arvanitoyannis I., Aiba S., Yamamoto N. (1997). Study of Biodegradability of Poly(d-valerolactoneco-L-lactide)s. J. Appl. Polym. Sci..

[13] Hiljanen-Vainio M., Karjalainen T., Seppälä J. (1996). Biodegradable lactone copolymers. I. Characterization and mechanical behavior of ε-caprolactone and lactide copolymers. J. Appl. Polym. Sci..

[14] Kricheldorf H.R., Berl M., Scharnagl N. (1988). Poly (lactones). 9. Polymerization Mechanism of Metal Alkoxide Initiated Polymerizations of Lactide and Various Lactones. Macrolactones.

[15] Kurcok P., Penczek J., Franek J., Jedlinski Z. (1992). Anionic Polymerization of Lactones. 14. Anionic Block Copolymerization of -Valerolactone and L-Lactide Initiated with Potassium Methoxide. Macromolecules.

[16] Fernández J., Etxeberria A., Sarasua J.R. (2016). Synthesis and properties of ω-pentadecalactone-co-δ-hexalactone copolymers: A biodegradable thermoplastic elastomer as an alternative to poly(ε-caprolactone). RSC Adv..

[17] Fernández J., Montero M., Etxeberria A., Sarasua J.R. (2017). Ethylene brassylate: Searching for new comonomers that enhance the ductility and biodegradability of polylactides. Polym. Degrad. Stab..

[18] del Río J., Etxeberria A., López-Rodríguez N., Lizundia E., Sarasua J.R. (2010). A PALS contribution to the supramolecular structure of poly(L-lactide). Macromolecules.

[19] Miguel O., Iruin J.J., Fernandez-Berridi M.J. (1997). Survey on transport properties of liquids, vapors, and gases in biodegradable poly(3-hydroxybutyrate) (PHB). J. Appl. Polym. Sci..

[20] Sangroniz A., Chaos A., Iriarte M., del Río J., Sarasua J.R., Etxeberria A. (2018). Influence of the rigid amorphous fraction and crystallinity on polylactide transport properties. Macromolecules.

[21] Sangroniz A., Sangroniz L., Aranburu N., Fernández M., Santamaria A., Iriarte M., Etxeberria A. (2018). Blends of biodegradable poly(butylene adipate-co-terephthalate) with poly(hydroxi amino ether) for packaging applications: Miscibility, rheology and transport properties. Eur. Polym. J..

[22] Sangroniz A., Sangroniz L., Gonzalez A., Santamaria A., Del Río J., Iriarte M., Etxeberria A. (2019). Improving the barrier properties of a biodegradable polyester for packaging applications. Eur. Polym. J..

[23] Ibbet R.N. (1993). NMR Spectroscopy of Polymers.

[24] De La Cal J.C., Leiza J.R., Asúa J.M. (1991). Estimation of reactivity ratios using emulsion copolymerization data. J. Polym. Sci. Part A Polym. Chem..

[25] Aubin M., Prud’Homme R.E. (1981). Preparation and properties of poly(valerolactone). Polymer.

[26] Olabisi O., Robeson L.M., Shaw M.T. (1979). Polymer-Polymer Miscibility.

[27] Paul D.R., Newman S. (1978). Polymer Blends.

[28] Sarasua J.R., Prud’Homme R.E., Wisniewski M., Le Borgne A., Spassky N. (1998). Crystallization and melting behaviour of polylactides. Macromolecules.

[29] Koros W.J. (1990). Barrier Polymers and Structures.

[30] Lizundia E., Vilas J.L., Sangroniz A., Etxeberria A. (2017). Light and gas barrier properties of PLLA/metallic nanoparticles composite films. Eur. Polym. J..

