# Supplementary material for: Lactide-Valerolactone Copolymers for Packaging Applications
Source: Polymers (Basel). 2021 Dec 23;14(1):52. doi: 10.3390/polym14010052 (PMC8747129; doi:10.3390/polym14010052)
Supplement: Supplementary file 1 [file polymers-14-00052-s001.zip › polymers-1486048-supplementary.pdf]

Supplementary Information

Lactide-valerolactone copolymers for packaging applications

Ainara Sangroniz, Leire Sangroniz, Shaghayegh Hamzehlou, Nora Aranburu, Haritz Sardon, Jose Ramon Sarasua, Marian Iriarte, Jose Ramon Leiza, Agustin Etxeberria

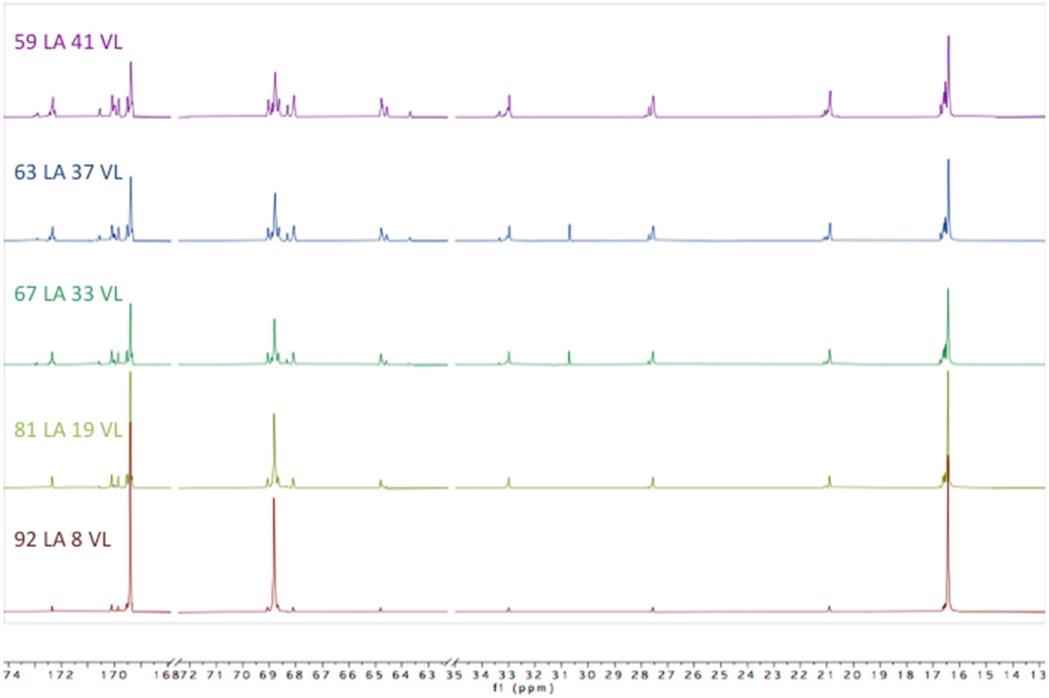

Figure S1. ^{13}C NMR of lactide-valerolactone copolymers.

Note S1. Estimation of the Monomer Reactivity Ratios

The reactivity ratios were estimated employing the evolution of the individual comonomer conversion over overall conversion employing the nonlinear method developed by de la Cal et al. [1].

The model is briefly described below, where lactide is denoted as LA and valerolactone as VL. The equations corresponding to LA conversion (x_{LA}) and overall conversion (X_T) are, in a batch reaction:

$$x_{LA} = \frac{[LA]_0 - [LA]}{[LA]_0} \quad \text{(S1)}$$

$$X_T = \frac{([LA]_0-[LA])+([VL]_0-[VL])}{[LA]_0+[VL]_0} \quad \text{(S2)}$$

where $[LA]_0$ and $[VL]_0$ are the initial concentration of LA and VL, respectively. Therefore;

$$dx_{LA} = -\frac{d[LA]}{[LA]_0} \quad \text{(S3)}$$

$$dX_T = \frac{-d[LA]-d[VL]}{[LA]_0+[VL]_0} \quad \text{(S4)}$$

$$\frac{dx_{LA}}{dX_T} = \frac{[LA]_0+[VL]_0}{[LA]_0}\frac{d[LA]}{d[LA]+d[VL]} \quad \text{(S5)}$$

Ring opening copolymerization of LA and VL is a chain polymerization thus, the *copolymer composition equation* [2,3] holds for this system:

$$\frac{d[LA]}{d[LA]+d[VL]} = \left(\frac{1+r_{LA}\frac{[LA]}{[VL]}}{2+r_{LA}\frac{[LA]}{[VL]}+r_{VL}\frac{[VL]}{[LA]}}\right) \quad \text{(S6)}$$

where r_{LA} and r_{Vl} are the reactivity ratios of LA and VL that are defined as,

$$r_{LA} = \frac{k_{pLALA}}{k_{pLAVL}} \quad \text{(S7)}$$

$$r_{VL} = \frac{k_{pVLVL}}{k_{pVLLA}} \quad \text{(S8)}$$

If Eq. S6 is replaced in Eq. S5 it gives:

$$\frac{dx_{LA}}{dX_T} = \frac{[LA]_0+[CL]_0}{[LA]_0}\left(\frac{1+r_{LA}\frac{[LA]}{[CL]}}{2+r_{LA}\frac{[LA]}{[CL]}+r_{CL}\frac{[CL]}{[LA]}}\right) \quad \text{(S9)}$$

Employing Equations S1 and S2, the ratio of monomer concentrations can be defined as a function of x_{LA} and X_T:

$$\frac{[LA]}{[VL]} = \frac{[LA]_0(1-X_{LA})}{[B]_0-X_T([LA]_0+[VL]_0)+[LA]_0x_{LA}} \quad \text{(S10)}$$

If Equation S10 is replaced in Equarion S9, lactide conversion can be integrated over the overall conversion. On the other hand, the cumulative composition can be described as a function of the individual conversion of LA, x_{LA}, and the overall conversion:

$$Y_{LA} = \frac{[LA]_0x_{LA}}{([LA]_0+[VL]_0)X_T} \quad \text{(S11)}$$

In order to calculate the reactivity ratios r_{LA} and r_{VL} a parameter estimation algorithm is employed that minimizes the objective function of Equation S12, H, where Y_{LAexp} is the cumulative composition refereed to LA determined by ^{1}H NMR experimentally and Y_{LAcal} is the cumulative composition calculated theoretically employing Equations S9-S11 and the initial monomer concentration. Subscripts i and j refer to the experiment and to the sample number of each experiments used in the estimation procedure. The only parameters of the model are the reactivity ratios.

$$H = \left[\sum_{i=1}^{N} \sum_{j=1}^{Pi} \left(Y_{LAexp} - Y_{LAcal}\right)^2\right] \quad \text{(S12)}$$

The model algorithm was coded in Matlab using ODE45 solver to solve ordinary differential equations and LSQNONLIN for nonlinear data fitting.

Table S1. Microstructure parameter of lactide-valerolactone copolymers.

VL % in the copolymer	l_{LA}	l_{VL}	R
8	12.27	1.12	0.97
19	4.81	1.15	1.07
33	2.44	1.21	1.24
37	2.10	1.24	1.28
41	1.82	1.27	1.34

Table S2. Thermal degradation of lactide-valerolactone copolymers: $T_{5\%}$, temperature at which 5 % of weight is lost, and $T_{50\%}$, temperature at which 50 % of weight is lost.

VL % in the copolymer	$T_{5\%}$ (ºC)	$T_{50\%}$ (ºC)
8	214.3	267.1
19	218.2	330.0
33	214.4	324.5
37	213.4	323.0
41	224.6	345.4

Table S3. Mechanical properties of lactide-valerolactone copolymers.

Sample	Tensile strength (MPa)	Young modulus (MPa)	Elongation at break (%)
PLLA	54.2 ± 4.0*	3390 ± 470	3.8 ± 0.8
8 VL	6.9 ± 2.5*	2050 ± 400	1.1 ± 0.4
19 VL	10.3 ± 1.4*	3020 ± 340	23.4 ± 4.8
	48.5 ± 6.2**		
33 VL	19.4 ± 3.9*	1150 ± 200	85.4 ± 13.2
	18.8 ± 4.1**		
37 VL	10.9 ± 1.5*	310 ± 60	210 ± 36
41 VL	0.73 ± 0.1*	4.3 ± 0.8	1800 ± 40

* Corresponds to ultimate stress

** Corresponds to yield stress

Table S4. Water vapour transmission rate and oxygen permeability of lactide-valerolactone copolymers.

Sample	*WVTR* (g mm m^{-2} day^{-1})	*P* O_2 (Barrer)
8 VL	6.80 ± 0.85	0.26 ± 0.02
19 VL	5.39 ± 0.67	0.19 ± 0.01
33 VL	7.89 ± 0.93	0.22 ± 0.02
37 VL	9.80 ± 0.64	0.28 ± 0.02
41 VL	11.54 ± 0.65	0.43 ± 0.06
